# Biodegradation of plastic polymers by fungi: a brief review

**DOI:** 10.1186/s40643-022-00532-4

**Published:** 2022-04-08

**Authors:** Munuru Srikanth, T. S. R. S. Sandeep, Kuvala Sucharitha, Sudhakar Godi

**Affiliations:** 1 Department of Biotechnology, College of Science and Technology, Andhra University, Visakhapatnam, 530003 India; 2 Department of Biotechnology, Pydah Degree College, Affiliated to Andhra University, Visakhapatnam, India; 3 Department of Human Genetics, College of Science and Technology, Andhra University, Visakhapatnam, 530003 India

**Keywords:** Biodegradation, Plastic polymers, Plastic degradation, Fungi, Degrading enzymes

## Abstract

Plastic polymers are non-degradable solid wastes that have become a great threat to the whole world and degradation of these plastics would take a few decades. Compared with other degradation processes, the biodegradation process is the most effective and best way for plastic degradation due to its non-polluting mechanism, eco-friendly nature, and cost-effectiveness. Biodegradation of synthetic plastics is a very slow process that also involves environmental factors and the action of wild microbial species. In this plastic biodegradation, fungi play a pivotal role, it acts on plastics by secreting some degrading enzymes, i.e., cutinase`, lipase, and proteases, lignocellulolytic enzymes, and also the presence of some pro-oxidant ions can cause effective degradation. The oxidation or hydrolysis by the enzyme creates functional groups that improve the hydrophilicity of polymers, and consequently degrade the high molecular weight polymer into low molecular weight. This leads to the degradation of plastics within a few days. Some well-known species which show effective degradation on plastics are *Aspergillus nidulans, Aspergillus flavus, Aspergillus glaucus, Aspergillus oryzae, Aspergillus nomius, Penicillium griseofulvum, Bjerkandera adusta, Phanerochaete chrysosporium, Cladosporium cladosporioides,* etc., and some other saprotrophic fungi, such as *Pleurotus abalones, Pleurotus ostreatus, Agaricus bisporus* and *Pleurotus eryngii* which also helps in degradation of plastics by growing on them. Some studies say that the degradation of plastics was more effective when photodegradation and thermo-oxidative mechanisms involved with the biodegradation simultaneously can make the degradation faster and easier. This present review gives current knowledge regarding different species of fungi that are involved in the degradation of plastics by their different enzymatic mechanisms to degrade different forms of plastic polymers.

## Introduction

Plastic is considered as one of the threatful elements in the environment because of its slow degradation in the environment which seriously takes some decades, so it’s considered a non-degrading material. These non-degradable plastics accumulated considered as solid waste on the earth's surface which is assumed as food by terrestrial animals, such as cows, buffaloes, and consuming it which causes the death of animals (Singh 2005). These plastics which form particulate matter by UV irradiation and weathering increase surface area and mobility and, therefore, easily incorporate into the food chain causing serious effects to all the living organisms (Bonhomme et al. 2003; Sen and Raut 2015). Disposing of the plastic waste in oceans leads accumulation of toxic chemicals, such as polychlorinated biphenyl (PCB’s), nonylphenol (NP), dichlorodiphenyltrichloroethane (DDT), polycyclic aromatic hydrocarbons (PAH), polybrominated diphenyl esters (PBDE), and bisphenol A (BPA) (Bryant et al. 2016), which have been found as a serious problem of indigestion, gastrointestinal blockages and reproductive problems in marine organisms. Due to this plastic pollution in the marine environment minimum of 267 species are being affected which includes sea turtles (86%) and seabirds (44%) (Coe et al. 1997). The worldwide annual production of non-degradable plastic ranges from 350 to 400 million tons out of that yearly 5–13 million tons of waste plastic are released into oceans which damages the ecological environment. They are various forms of plastics, i.e., nylon, polycarbonate, polyethylene terephthalate, polyethylene, polypropylene, polystyrene, polytetrafluoroethylene, polyurethane, and polyvinyl chloride (Usha et al. 2011). To degrade these plastics, there are different methods, such as photodegradation, thermo-oxidative degradation, hydrolytic degradation, and biodegradation. Photodegradation and thermo-oxidation are categorized under abiotic degradation, whereas biotic degradation involves the action of microbes. Photodegradation involves continuous exposure of UV light from the sun or artificial source on plastic material which eventually incorporates oxygen molecules in between the structure which leads to breaking the complex polymers into simple molecules, while thermo-oxidative involves exposure of heat on the plastic polymers (Geweret et al. 2015). However, exposure to high temperatures leads burning of plastic produces toxic gases into the environment and poses health hazards by causing lung diseases and cancer after inhalation (Pramila and Vijaya Ramesh 2011). Compared with other degradations, the biodegradation method is mostly preferred due to its pollution-free mechanism and eco-friendly process. In biodegradation, the process is initiated by micro-organisms, i.e., bacteria and fungi. In general, this biodegradation of plastics involves the growth of fungi on the surface of plastic, where plastic is consumed as a food source by the fungi under the influence of environmental factors, such as temperature and pH. These fungi will secrete enzymes, such as cutinase, lipase, and proteases, carboxylesterases, esterases, lignocellulolytic enzymes, and some pro-oxidant ions which will degrade the plastics. By oxidation/hydrolysis enzyme improves the hydrophilicity of polymers and consequently degrade the high molecular weight polymer into low molecular weight. As high molecular weight is a large compound that cannot be transported across the cellular membrane of the fungi thus it primarily depolymerizes it into small monomers before they cross the cell membrane (Shah et al. 2008). The enzyme activity is mainly dependent on the solvent properties and the enzyme activity increases with polarity and decreases with the viscosity of the solvent in the biodegradation of polymers (Patel et al. 2013). The process becomes more effective when photodegradation and thermo-oxidative degradation is followed by biodegradation, because as by photodegradation and thermo-oxidative degradation, the plastic debris will be broken-down from complex to simple material so biodegradation on such material will be easy and does not require much time. Even practicing different methods, plastic degradation takes sufficient time to complete the process. The best solution for decreasing this plastic pollution is using biodegradable plastics. These biodegradable polymers are designed to degrade quickly by the microbes due to their ability to degrade organic and inorganic materials, such as lignin, starch, cellulose, and hemicelluloses (Kumar et al. 2013). In this review, we described various fungi involved in the biodegradation of different types of plastic polymers and summarized recent studies on enzymes that are produced by various fungi for the biodegradation of plastics. In additional, we discussed the effective degradation of fungi on selective plastics and plastic degradation by edible fungi.

### Enzymes that involve in plastic degradation

#### Cutinases

Cutinases are a subclass of esterase enzyme which is identified by their ability to hydrolyze polyesters with high molar mass (Chen et al. 2013). They are α/β hydrolases or carboxylic ester hydrolases which were observed in plant pathogenic fungi, i.e., *Fusarium solani pisi* (Kolattukudy and Brown 1975; Kolattukudy et al. 1981; Heredia 2003). Cutinases are produced by *Fusarium solani* (Alisch-Mark et al. 2006; O’Neill et al. 2007), *Penicillium citrinum* (Liebminger et al. 2007), *Pichia pastoris (*Munari 2019) *Aspergillus oryzae*, *Humicola insolens*. The activities of *Fusarium solani pisi* cutinase (FsC) and *Humicola insolens* cutinase (HiC) were shown to be capable of degrading low crystallinity PET film with 97% weight loss being observed within 96 h (Ronqvist et al. 2009). PET-hydrolases belong to the cutinases group which also has promising results in the biodegradation of PET. An enzyme that is similar to cutinase in function, i.e., isolated from *Cryptococcus* sp. Strain S-2 was identified for its effective degradation of high molecular weight plastic, i.e., polylactic acid (PLA) based plastic (Gemeren et al. 1998). Cutinase 2p from *Arxula adeninivorans* showed enzymatic decomposition (hydrolysis/oxidation) of electrospun polycaprolactone fiber mats (Furukawa et al. 2019).

#### Lipases

Lipases are enzymes that catalyze the hydrolysis of lipids they are also the subclass of esterases enzyme. Some fungal species that are well known to produce lipases and are involved in the degradation of plastics, i.e., *Rhizopus delemer, Candida antarctica *(Vertommen et al. 2005), *Thermomyces lanuginosus* (Eberl et al. 2009), *Candida rugosa* were degrading poly (butylene succinate-*co*-hexamethylene succinate) copolymer. (Pereira et al. 2001). *Rhizopus delemer* lipase degraded 53% of the polyester type-polyurethanes (ES-PU) film after 24 h reaction (Tokiwa and Calabia 2009). A lipase enzyme extracted from the yeast *Cryptococcus* sp, exhibited hydrolysis of polybutylene succinate (PBS) and polybutylene succinate-*co*-adipate (PBSA) (Thirunavukarasua et al. 2008). Lipase B from *Candida antarctica* was effectively hydrolyzing PET to TPA (Carniel et al. 2017), lipase FE-01 from *Thermomyces languinosus* showed enzymatic decomposition of electrospun polycaprolactone fiber (Furukawa et al. 2019).

#### Proteases

Proteases are enzymes that cleave the long peptide chain to short peptides or break down proteins to polypeptide chains by hydrolysis this process is termed to be proteolysis. *Aspergillus, Trichoderma, Paecelomyces, Penicillium, Alternaria, Fusarium* (Loredo-Treviño et al. 2011; Cosgrove et al. 2007), *Phaenarochete* (Shimao 2001), *Pestalotiopsis* (Russell 2011) *Rhizopus, Mucor, Humicola, Thermoascus, Thermomyces* (Souza et al. 2015) are some of the important fungal species which are producing proteases to degrade plastics. *Anthrobotrys oligospora* synthesis serine protease that can degrade polylactic acid (Ozsagiroglu et al. 2012).

#### Esterases

Esterases are hydrolase enzymes that split esters into alcohols and acids by the addition of water molecules. Esterases are also involved in plastic degradation which is produced by both bacteria and fungi, esterase from *Comamonas acidovorans* is helpful in the degradation of low molecular weight PLA (plastic obtained from renewable resources). *Purpureocillium lilacinum* and *Curvularia senegalensis* are a group of fungi that degrade poly (butylene succinate-*co*-adipate) and polyurethane (Yamamoto-Tamura et al. 2015). *Aspergillus flavus, Aspergillus tubingensis *are assumed that they secrete esterases that are responsible for the degradation of plastics (Khan et al. 2017; Tokiwa et al. 2009). Esterase, derived from *Xepiculopsis graminea*, and *Penicillium griseofulvum* reported degrading polyurethane (Brunner et al. 2018). Lipase and esterase also degrade polycaprolactone polymers (Ganesh et al. 2017).

#### Laccase

Laccases are multi-copper oxidases that catalyze the oxidation of phenolic compounds. It utilizes molecular oxygen as a co-substrate and produces water and by-products (Nunes and Kunamneni 2018). The special ability of these laccases is they oxidize lignin so they involve in degrading lignin (Osma et al. 2010). Laccase can also involve in the oxidation of the hydrocarbon backbone of polyethylene (Sivan 2011). *Cochliobolus sp*., a specific fungus that is degrading PVC by laccases (Sumathi et al. 2016). *Bjerkandera adusta* TBB-03 was identified for its ability to degrade HDPE under lignocellulose substrate treatment by laccase production (Bo Ram Kang 2019). *Trametes versicolor, Pleurotus ostreatus, Streptomyces, P. ostreatus and T. pubescens* produce laccase that degrades polyethylene (Osma et al. 2010). In general, the Ligninolytic enzyme families include phenol oxidase (laccase), heme peroxidases, lignin peroxidase (LiP), manganese peroxidase (MnP), and versatile peroxidase (VP) (Dashtban et al. 2010). Papain and urease are the two proteolytic enzymes that were found to degrade medical polyester polyurethane by hydrolysis of urethane and urea linkages by producing free amine and hydroxyl groups (Phua et al. 1987). Penicillium-derived laccase potentially involves in PE breakdown (Abd El-Rehim et al. 2004).

#### Peroxidases

Peroxidases are enzymes that fall under the oxidoreductase class which catalyzes oxidation–reduction reactions by the action of free radicals on compounds to form oxidized and polymerized compounds. They are also called catalases; peroxidases of fungi are regarded to be more efficient in converting lignin. This class includes lignin peroxidases (LiP), manganese peroxidases (MnP), and versatile peroxidases (VP) which are mostly found in white-rot fungi (Hofrichter and Ullrich 2006). *Phanerochaete chrysosporium*, and *Trametes versicolor* showed effective degradation of high-molecular-weight polyethylene, where MnP/Manganese peroxidases is the key enzyme in polyethylene degradation (Iiyoshi et al. 1998). *Phanerochaete chrysosporium, Pleurotus ostreatus* and S22 showed a high capacity for lignin degradation at pH 9.0–11. Ligninolytic enzymes, includes LiP, MnP, and laccase (Wu et al. 2005). *Fusarium graminearum* showed polyethylene degradation by producing peroxidase (Ganesh et al. 2017). *Aspergillus flavus, Aspergillus niger, and Fusarium graminearum* produce manganese peroxidase, lignin peroxidase and these enzymes induce the biodegradation of PCB (polyethylene carry bag). The maximum range of lignin peroxides are produced in *Aspergillus niger* and *Aspergillus flavus* (Bholay et al. 2012).

#### Pro-oxidant ions

Pro-oxidant ions are chemical elements that induce oxidative stress by releasing reactive oxygen species or by inhibiting the antioxidant system. These elements can be as transition metals, such as Fe, Co (Weiland et al. 1995), and Mn (Jakubowicz 2003), and can enhance the photo- and thermo-oxidation, PE chains may lead to radical reactions that lead to cleavage in polymer chains (Koutny et al. 2006). The pro-oxidants are transient metal ions, added in form of stearate or other organic ligand complexes, mostly stearates of Fe^3+^, Mn^2+^ (Jakubowicz 2003), or Co^2+^ (Weiland et al. 1995). Fe^3+^ complex involves in the photo-oxidation process as a source of radicals for reaction initiation, the Mn^2+^ or Co^2^ is necessary for oxidation without the influence of light, when they catalyze the decomposition of peroxides associated with chain cleavage. It was identified that trace amounts of metals such as Co, Mn, Fe, Cu, and Ni showed increasing the rate of oxidation (Gorghiu et al. 2004), these facilitate cleavage of molecules into smaller fragments containing hydrophilic oxygenated groups that can be easily degraded by microbes (Shang et al. 2003).

### Enzymatic mechanisms involved in plastic biodegradation by fungi

**Fig. 1:**
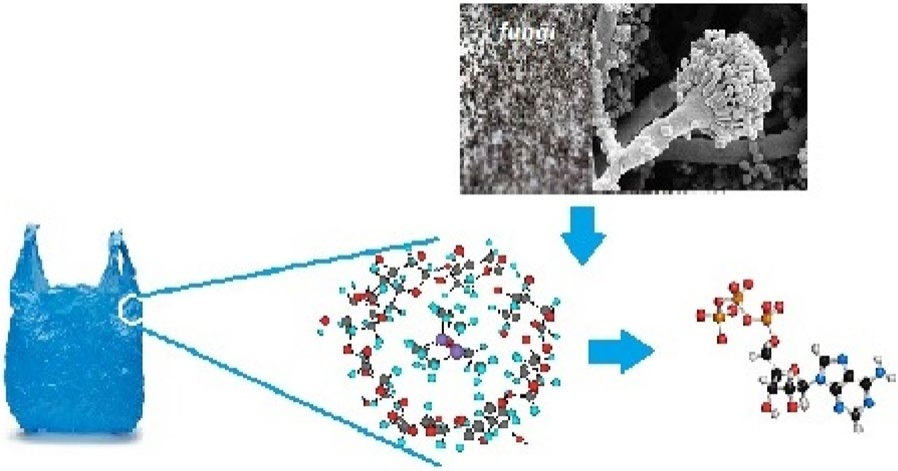
Complex polymer chains of polythene are converted to simple polymer chains by action of fungi (biodegradation)

The mechanism of biodegradation involves the action of microbial enzymes on the surface of the plastics. The microbes such as bacteria and fungi attach to the plastic film and inert the enzymes and grow on it by utilizing it as substrate and source of nutrition. Therefore, the polymers slowly get depolymerized and degradation will be compiled by mineralization process, where H_2_O (water), CO_2_ (carbon dioxide), CH_2_ (methane) are end products (Frazer 1994; Montazer et al. 2019). The ability of fungi was they can invade substrates using enzymes that can detoxify pollutants. Fungi can also produce some surface-active proteins, i.e., hydrophobins to coat hyphae to hydrophobic substrates. The growth of many fungi can also cause small-scale swelling and bursting, as the fungi penetrate the polymer solids (Griffin 1980). The degradation of plastics by some fungi occurs through the intracellular and extracellular enzymatic systems. The intracellular enzymatic system acts as an internal mechanism for detoxification and plays a major role in fungal adaption (Jeon et al. 2016; Olicón-Hernández et al. 2017; Schwartz et al. 2018; Shin et al. 2018). This system is mediated by the cytochrome P450 family (CYP), Phase I enzyme epoxidase and, Phase II enzyme transferases which involve oxidation and conjugation reactions. Cytochrome P450 family are heme-containing mono-oxygenases that are involved in catalyzing various enzymatic reactions (Shin et al. 2018). Cytochrome P450 enzymes are important for primary metabolism, enabling protection of the hyphal wall integrity and the formation of the spore outer wall (Črešnar and Petrič 2011). CYP isoforms are anchored in the membrane of the endoplasmic reticulum, having their active sites connected to both the cytosolic and membrane environments so they can uptake substrate from both surroundings (Šrejber et al. 2018). CYP contains three cofactors (NADPH^+^, H^+^, FAD, and Heme) and two enzymes (NADPH: CYP reductase and cytochrome P-450 hydrolase). The extracellular enzymatic system consists of a hydrolytic system that produces hydrolases that are involved in polysaccharide degradation and the unspecific oxidative system involved in breaking down complex structures, such as lignin degradation (Sánchez 2009). The unspecific oxidative system can oxidize a wide range of substrates. It is formed mainly by nonspecific oxidoreductases, including enzymes, such as class II peroxidases (manganese peroxidase, lignin peroxidase, and versatile peroxidase), laccases, and unspecific peroxygenases. These enzymes transfer electrons from organic substrates to molecular oxygen (laccases) by oxidation–reduction reactions using H_2_O_2_ as an electron-accepting co-substrate or by epoxidation, aromatic preoxygenation, and sulfoxidation (Karich et al. 2017). This enzymatic complex is produced mainly by wood-degrading fungi, such as basidiomycetes (Sánchez 2009). The action of fungi on the surface of plastics can be affected by environmental factors such as moisture, pH, temperature, etc. sufficient moisture is required for activation of fungi, appropriate pH environment is required for the action of enzymes on plastic polymer and equally, temperature plays a vital role in this biodegradation process, polymers of high melting point take more time to degrade than polymers of low melting point (Fig. 1).

### Aerobic and anaerobic plastic biodegradation

In aerobic biodegradation, the degradation involves in presence of oxygen which is also known as aerobic respiration. Aerobic fungi use oxygen as an electron acceptor, and breakdown complex organic compounds into smaller organic compounds often producing co2 and water as end products (Seymour et al. 1899), formation of carbon dioxide (Sturm test) are good indicators for polymer degradation and are the most often used methods to measure biodegradation in laboratory tests. Anaerobic degradation occurs in absence of oxygen. Degradation of complex polymers into smaller units by microbes with CH_2_, CO_2_, H_2_O, and biomass as their by-products. Anaerobic fungi in the absence of oxygen utilize other sources as their electron acceptor, such as sulfate, nitrate, iron, manganese, and carbon dioxide for biodegradation (Alshehrei 2017). Two *Pestalotiopsis microspora* isolates were observed to grow on PUR as the sole carbon source under both aerobic and anaerobic conditions, effective degradation activity was observed under anaerobic growth using PUR (Jonathan and Russell 2011).

### Biodegradation of various plastics

Polythene is mostly used plastics in daily life due to its easy processing for products, such as plastic bags, plastic films, packing food materials, textiles (Arutchelvi et al. 2021). Polyethylene is chemically represented as (C_2_H_4_) n. PE is a combination of polymers of ethylene with different values of n. They are low-density polyethylene and high-density polyethylene. Low-density polyethylene is processed by applying high pressure (1000–5000 atm) and high temperature (520 kelvins), whereas high-density polyethylene requires low pressure (6–7 atm) and low temperature (333–343 K) (Lee et al. 1991). It is reported that Polyethylene constitutes 64% of total synthetic plastics as it is been used for manufacturing bottles, carry bags, disposable articles, garbage containers, margarine tubs, milk jugs, and water pipes. Annually 500 billion to 1 trillion polythene bags are being used daily all over the world. The usage of polythene is increased at a rate of 12% annum and approximately 140 million tonnes of synthetic plastic polymers are produced worldwide annually (Roy et al. 2008). *Phanerochaete chrysosporium* is fungal species that degrade high molecular weight polyethylene under nitrogen-limited and carbon-limited conditions (Shimao 2001). *Aspergillus, Cladosporium, Fusarium, Penicillium, Phanerochaete* have been reported for polyethylene degradation (Danso et al. 2019; Glaser 2019; Restrepo-Florez et al. 2014). Additives free Polyethylene degradation was identified in *Pencillium. simplicissimum* (Esmaeili et al. 2013), *Aspergillus niger, Aspergillus japonicas and Fusarium. sp* (Raaman et al. 2012). *Penicillium chrysogenum* NS10 (KU559907), *Penicillium oxalicum* NS4 (KU559906) were identified for degrading HDPE and LDPE (Ojha et al. 2017).

### Polyethylene terephthalate/PET

Polyethylene terephthalate is a semicrystalline, thermoplastic, strong and durable, chemically and thermally stable, has low gas permeability, and is easily processed. PET is used as fibers, sheets and films, electronics, automotive parts, houseware, lighting products, power tools, sports goods, photographic applications, X-ray sheets and textiles, and in food and beverage packaging (especially, soft-drink and water bottles) (Awaja and Pavel 2005; Kint and Muñoz-Guerra 1999; Levchik and Weil 2004; Bergeret et al. 2009). Polyethylene and polypropylene represent about 92% of the synthetic plastics produced, and they are used for the production of plastic bags, disposable containers, bottles, packaging materials, etc. (Byuntae et al. 1991). The enzymes involved in the degradation (e.g., PET hydrolase and tannase, MHETase) are typical serine hydrolases, such as cutinases, lipases, and carboxylesterases (Wei et al. 2014). Fungal cutinases of *Fusarium* and *Humicola* were identified for their degradation in PET. Even lipase CalB from *Candida antarctica* was also used in the PET degradation process (Carniel et al. 2017). *Aspergillus sp., Penicillium sp., and Fusarium sp* are used as biological agents to degrade PET and PS foam (Umamaheswari and Murali 2013). *Aspergillus oryzae, C. antarctica, and Penicillium citrinum* are among other fungal enzymes that have been investigated for activity on PET (Zimmermann and Billig 2010; Kawai et al. 2019).

### Polypropylene/PP

Polypropylene is also referred to as polypropene, a thermoplastic polymer. Polypropylene belongs to partially crystalline polyolefins, mostly used polypropylene is isotactic. PP is used in manufacturing rugs, mats, carpets, ropes, and chairs. It is also used in manufacturing laboratory-required equipment, such as wash bottles, centrifuge tubes, Eppendorf tubes, tips for pipettes, etc. The surfaces of polypropylene are hydrophobic, because it has CH and CH2 groups along its backbone and a CH_3_ pendant group. To make the polymer surface more hydrophilic, pre-treatments such as UV radiation, gamma sterilization, or thermal treatments are followed (Koutny et al. 2006; Halina et al. 2005; Alariqi et al. 2006). *Bjerkandera adusta* (Butnaru et al. 2016), *Lasiodiplodia theobromae* (SanaSheik et al. November 2015), *Coriolus versicolor,* (Kord et al. 2021) fungi are identified for their ability in the degradation of Polypropylene.

### Polyvinylchloride/PVC

Polyvinylchloride is a strong polymer, composed of repeating chloroethyl units (Fischer et al. 2014), these are low cost, and show biological and chemical resistance. PVC comes in two forms, i.e., rigid and flexible. Pure PVC is soluble in tetrahydrofuran and insoluble in alcohols. PVC is similar to the structure of chlorophenol compounds. Compared with PET and PS, polyvinylchloride (PVC) is considered to be a hard plastic for biodegradation. Some fungal species which showed PVC degradation are *Cochliobolus sp*. (Sumathi et al. 2016), *Phanerochaete chrysosporium, Aspergillus niger* (Ali et al. 2014), *Penicillium funiculosum ATCC 9644, Trichoderma viride ATCC 13631, Paecilomyces variotii CBS 62866, Aspergillus niger (ATCC 6275)* (Whitney 1996), *Aureobasidium pullulans* (Webb et al. 2020), *Chaetomium globosum (ATCC 16021)* (Vivi et al. 2018). Some yeast-like fungi *Rhodotorula aurantiaca and Kluyveromyces spp* also showed some degrading properties towards polyvinylchloride.

### Polystyrene (PS)

Polystyrene is a thermoplastic polymer, aromatic hydrocarbon polymer which is composed of monomers, i.e., styrene (John Scheirs 2003). They are in the form of solid or foamed, its chemical formula is (C_8_H_8_) n. PP is degraded by acetone, chlorinated solvents, and aromatic hydrocarbon solvents. It is used for protective packaging such as packing food items and jewel cases and used for manufacturing cases for CDs, DVDs, containers, lids, bottles, trays, tumblers, etc. Degrading polystyrene ability was identified in *Cephalosporium spp*. and *Mucor spp. Gloeophyllum striatum DSM 9592* and *Gloeophyllum trabeum DSM 1398* strains causing almost 50% reduction in molecular weight of polystyrene. White rot fungi *Pleurotus ostreatus, Phanerochaete chrysosporium*, and *Trametes versicolor*, and the brown-rot fungi *Gloeophyllum trabeum* were capable of depolymerization of polystyrene when coincubated together with lignin (Krueger et al. 2015; Milstein et al. 1992). *Aspergillus sp., Penicillium sp.,* and *Fusarium sp. *were also identified for their ability to degrade PET and PS foam (Umamaheswari and Murali 2013) *Curvularia species* hyphae had adhered to and penetrated the polymer’s structure (Motta et al. 2009). *Geomyces, Mortierella* species also involve in degrading polystyrene (Oviedo-Anchundia et al. 2021).

### Polyurethane/PUR

Polyurethane is a polymer composed of organic units which are joined by carbamate (urethane) links. They are in rigid and flexible foam forms, varnishes and coatings, adhesives, electrical compounds, and fibers, such as spandex and polyurethane laminate (Gama et al. 2018). Polyurethanes are a type of plastic that has wide use in industries, they are synthesized from polyols and polyisocyanates. This polyurethane is degraded by the following fungi, i.e., *Gliocladium roseum, Aspergillus spp., Emericella spp., Fusarium spp., Penicillium spp., Trichoderma spp., Gliocladium pannorum, Nectria gliocladiodes, Penicillium ochrochloron, Aureobasidium pullulans, Rhodotorula aurantiaca, and Kluyvermyces spp* (Cosgrove et al. 2007; Lagauskas and Pečiulytė 2009; Webb et al. 2000). Some fungi such as *Plectosphaerella, Nectria, Neonectria, Phoma, and Alternaria* also showed their ability for PU biodegradation. *Aspergillus niger* was also identified for its quite slow growth with visible signs of deterioration occurring only after 30 days (Russell 2011). *Pestalotiopsis microspora*, utilizing polyurethane as a carbon source with an enzyme serine hydrolase and degrade it within a few days (Russell 2011). *Comamonas acidovorans* produce polyurethane esterase that degrades polyurethane and low and high molecular weight polylactic acid (Bhardwaj et al. 2012). In degradation of polyurethane, proteases are more effective than esterases (Ozsagiroglu et al. 2012). Recent studies identified *Candida ethanolica* (Zafar et al. 2013), and *Candida rugosa* (Gautam et al. 2007) as PUR degraders. *Cladosporium pseudo cladosporioides, Cladosporium tenuissimum, Cladosporium asperulatum, Cladosporium montecillanum, Aspergillus fumigatus, Penicillium chrysogenum* were also reported for degrading PUR (Álvarez-Barragán et al. 2016; Mathur and Prasad 2012).

### Polycarbonate/PC

Polycarbonate is a thermoplastic polymer that contains carbonate groups (−O−(C=O). its rigid and strong polymer. It is used in manufacturing electronic components such as TVs screens computer screens, compact discs, DVDs, automotive, aircraft, and some security elements such as bulletproof sheets, eyeglasses/lenses. Degradation of polycarbonates is typical in-process due to its rigid structure and usually takes years. *Phanerochaete chrysosporium* NCIM 1170 a white-rot fungus showed degrading properties towards polycarbonates (Artham and Doble 2010). *Geotrichum* spp., are identified by their aryl alcohol oxidases and Mn^2+^-oxidizing peroxidase enzymes and degrading polycarbonates (Romero et al. 2007). Some common fungal species which are showing biodegradation of polycarbonates are *Fusarium,* *Ulocladium, Chrysosporium*, and *Penicillium* (Arefian et al. 2013).

### Plastic polymer biodegradation by edible fungi

Edible fungi/Edible Mushrooms which are considered macrofungi are fleshy and fruit bodies which is rich in their nutritional benefits. They are artificially cultivated on a suitable substrate, such as straw, husk, sugarcane residues, leaves, etc. The mushrooms grow on the surface of the substrate by absorbing the nutrients from the substrate. In the place of other substrates, plastic films/sheets are used as substrate. Some important fungi species which showed their ability to absorb nutrients from the plastic polymers they are *Pleurotus abalones, Pleurotus ostreatus, Agaricus bisporus* these species are by secretion of enzyme laccases utilized the polyethylene and polystyrene as carbon sources and showed growth by degrading the plastics. (Hock et al. 2019). *Pleurotus eryngii, Lentinula edodes*, fungi showed the ability to degrade BPA (Bisphenol-A) and DEHP (di-2-ethyhexylphthalate) plastics by secretion of manganese peroxidase (MnP) enzyme (Hock et al. 2019). The capability of degrading green polyethylene and oxo-biodegradable (D2W) plastics without prior physical treatment was observed in *Pleurotus ostreatus*. The formation of mushrooms from the plastic as a substrate is a very new approach to control plastic pollution, and the productivity of mushrooms can be increased by altering the composition of the substrate (Luz et al. 2013).

## Conclusions

The increase in plastic pollution greatly affects living organisms, biodegradation of plastics by fungi can help to decrease the problem. Biodegradation of plastic polymers has been one of the current focussed areas of research on solving plastic pollution. The review provides eminent information on various fungi which are involved in degrading different types of plastic polymers, and specific degrading enzymes produced by various fungi which are involved in the biodegradation mechanism. Though many studies identified the degrading abilities in fungi very few shows effective biodegradation. Genetic engineering could be a preferred strategy to enhance the ability of fungi in biodegradation of plastic polymers. Several strategies, such as random mutagenesis and site-directed mutagenesis, genome editing, advanced computational modelling, computational genomics, are the recent strategies to address enzyme engineering. In addition, genetically engineered robust enzyme systems could be an effective strategy to reduce plastic waste.

## Data Availability

Not applicable.
